# The oxidative damage initiation hypothesis for meiosis

**DOI:** 10.1007/s00497-013-0234-7

**Published:** 2013-08-31

**Authors:** Elvira Hörandl, Franz Hadacek

**Affiliations:** 1 Department of Systematic Botany, Albrecht-Haller-Institute for Plant Sciences, Georg-August-University of Göttingen, Untere Karspüle 2, 37073 Göttingen, Germany; 2 Department of Plant Biochemistry, Albrecht-Haller-Institute for Plant Sciences, Georg-August-University of Göttingen, Justus-von-Liebig-Weg 11, 37077 Göttingen, Germany

**Keywords:** Apomixis, DNA repair, Meiosis, Oxidative stress, Paradox of sex

## Abstract

The maintenance of sexual reproduction in eukaryotes is still a major enigma in evolutionary biology. Meiosis represents the only common feature of sex in all eukaryotic kingdoms, and thus, we regard it a key issue for discussing its function. Almost all asexuality modes maintain meiosis either in a modified form or as an alternative pathway, and facultatively apomictic plants increase frequencies of sexuality relative to apomixis after abiotic stress. On the physiological level, abiotic stress causes oxidative stress. We hypothesize that repair of oxidative damage on nuclear DNA could be a major driving force in the evolution of meiosis. We present a hypothetical model for the possible redox chemistry that underlies the binding of the meiosis-specific protein Spo11 to DNA. During prophase of meiosis I, oxidized sites at the DNA molecule are being targeted by the catalytic tyrosine moieties of Spo11 protein, which acts like an antioxidant reducing the oxidized target. The oxidized tyrosine residues, tyrosyl radicals, attack the phosphodiester bonds of the DNA backbone causing DNA double strand breaks that can be repaired by various mechanisms. Polyploidy in apomictic plants could mitigate oxidative DNA damage and decrease Spo11 activation. Our hypothesis may contribute to explaining various enigmatic phenomena: first, DSB formation outnumbers crossovers and, thus, effective recombination events by far because the target of meiosis may be the removal of oxidative lesions; second, it offers an argument for why expression of sexuality is responsive to stress in many eukaryotes; and third, repair of oxidative DNA damage turns meiosis into an essential characteristic of eukaryotic reproduction.

## Introduction: the paradox of sex

Sex is paradoxical, enigmatic, and still regarded as a major unresolved problem in evolutionary biology (Otto 2009). In eukaryotes, it involves meiosis–mixis cycles and is tied to reproduction. The prevalence of sex in eukaryotes is striking because of the high costs alleged to it (Bell 1982; Maynard Smith 1978). Recombination during meiosis breaks up beneficial gene combinations. Associated with these processes are the risks of errors and mismatches during pairing of homologous chromosomes, plus the time needed for meiosis (Lewis 1987). A further hint to costs of sex emerged only in the last 1–2 decades from a better understanding of functional backgrounds: a set of about 50 proteins is involved that must act in a concerted manner to make meiosis work despite a great risk of failure and sterility occurring even in mutants of single genes (Richardson et al. 2004). Mixis, which requires two parental individuals for conducting fertilization, merging of cells and genomes, adds further costs (Lewis 1987): mate searching, mate finding, sexual selection, competition for mating partners, and finally physical contact damage. Mixis, however, has many specific features in the various groups of eukaryotes, such as distribution of genders, and its costs must be considered differentially in the respective eukaryotic kingdoms (Charlesworth 1980; Lewis 1987).

Meiosis, in contrast, is the main shared feature for eukaryotic sex. In fact, the problem of the maintenance of sex can be largely addressed by the question, “For what good is meiosis?” Meiosis genes and the meiosis protein machinery are highly conserved across eukaryotes (Malik et al. 2008; Ramesh et al. 2005). Meiosis differs from mitosis in only a few steps and may have evolved in early eukaryotes out of mitosis (Wilkins and Holliday 2009) or even together with mitosis (Cavalier-Smith 2010). About 20 major hypotheses have been proposed to explain the paradox of sex. Three major theories attempt to explain the function of meiosis (see Birdsell and Wills 2003; for detailed review): (1) Meiotic sex is the mechanism for creating recombination and thus new gene combinations in the offspring, which likely increases the adaptive potential of genetically variable offspring; (2) Meiosis is a phylogenetically conserved feature that cannot be eliminated because of the ancestral fixation of meiosis–mixis cycles; (3) Meiosis is a restoration tool for prevailing the integrity of nuclear DNA by repairing DNA lesions, by eliminating deleterious mutations, and by repair of epigenetic damage. None of these theories, however, provides an all-inclusive answer for the paradox of sex (Birdsell and Wills 2003). It was recognized in the 1980s that theories based on the benefits of genetic variation are problematic for various reasons: (1) recombination is not necessarily beneficial as also positive gene associations can be lost; (2) sex does not necessarily result in recombination; (3) genetic variation is a group advantage that brings no immediate benefit to individuals that bear the costs of sex. For detailed reviews on these classical models the reader is referred to Birdsell and Wills (2003), Otto (2009), and Hörandl (2009).

The model of fitness-associated recombination (FAS) postulates that an organism invests more into sexual reproduction in environments where it is maladapted and the fitness of the organism is low (Hadany and Otto 2009; Otto 2009). The model takes into account that many facultatively sexual eukaryotes (yeasts, algae) show more sex under stress conditions (Schoustra et al. 2010). However, it is difficult to apply this model of condition-dependent sex to organisms with obligate sexual reproduction, and a functional model for the control mechanisms of meiosis under the FAS model is still missing.

Recent research has confirmed that meiosis is highly conserved in eukaryotes (Ramesh et al. 2005; Malik et al. 2008), but the question remains as to why such a costly mechanism without a clear benefit has not been eliminated by selection (Birdsell and Wills 2003). The repair hypothesis has gained support from experimental studies (Nedelcu and Michod 2003; Nedelcu et al. 2004). Recently, Hörandl (2009) proposed as an alternative a combinational theory that sex is altogether a comprehensive DNA restoration mechanism by combining the DNA repair function during meiosis and selective elimination of defect mutants in the haploid phase.

The alternative, asexual reproduction, is thought potentially to avoid some costs of sex (Bell 1982; Maynard Smith 1978). Nevertheless, asexual reproduction is rare in general and appears scattered on terminal nodes of phylogenies (Hörandl and Hojsgaard 2012; Simon et al. 2003). In almost all multicellular eukaryotes, asexual reproduction only modifies meiosis–mixis cycles; these modifications do not follow a general scheme but vary in different steps. Animals, for instance, often reproduce via automixis that still involves meiosis, but finally meiotic products fuse to form a diploid cell that develops parthenogenetically (Engelstädter 2008). Cytologically, automixis is similar to autogamy in plants where female and male meiosis produce spores separately in the respective organs of the same plant; gametogenesis proceeds normally, and then, just the gametes of the same individuals fuse again (self-fertilization). Automictic and autogamous organisms avoid the costs of a mating partner, but they do not skip meiosis. In these cases, meiosis even reduces genetic diversity by increasing homozygosity and the risk of inbreeding depression and loss of complementation (Archetti 2004). Animals with cyclical parthenogenesis, like *Daphnia,* have an almost complete set of meiosis genes but show only expansions of copy number and differences in gene expression. Cytologically, parthenogenetic reproduction differs from sex in only a few steps during meiosis, specifically, altered sister chromatid cohesion, lack of interhomolog cohesion, and different kinetochore attachment that result in diploid egg cells (Schurko and Logsdon 2008).

Apomixis in land plants also represents only an alteration of the sexual meiosis–mixis cycles. In sexual plants, meiosis generates haploid spores that develop into gametophytes that produce gametes, and fusion of gametes results in a zygote that develops into the sporophyte. Apomixis in flowering plants basically combines two developmental alterations of female sexual development: first, the bypass or alteration of meiosis that is still present (apomeiosis), and second, the development of an unfertilized egg cell into an embryo (parthenogenesis). This combination can be achieved in two ways. Gametophytic apomixis results in an unreduced gametophyte (embryo sac) either from an unreduced megaspore mother cell after a restitutional meiosis (diplospory) or from a somatic cell in the nucellus (apospory). Aposporous initials of embryo sacs often arise in parallel to meiotic products and replace megaspores during gametophyte development. The unreduced egg cell develops parthenogenetically into an embryo. Sporophytic apomixis, in contrast, starts the formation of an embryo directly from an unreduced cell of the ovule, either from a nucellus cell (nucellar embryony) or from the integument (integumentary embryony). Since such embryos usually arise in parallel to sexually formed embryos, this form of apomixis is also designated as adventitious embryony. Finally, the formed seeds are comprised of both sexual and apomictic embryos (polyembryony). The fertilization of the polar nuclei (pseudogamy) is retained in c. 90 % of apomicts (Mogie 1992), while pollen-independent endosperm development is rare (autonomous apomixis). Pollen is, therefore, at least partly functional. Male meiosis and microsporogenesis are maintained in apomictic plants without any fundamental change; disturbances of male meiosis are usually seen only as consequences of hybrid and/or polyploid origin (Asker and Jerling 1992). Uniparental reproduction is possible for pseudogamous apomicts because they are usually self-fertilizing (Hörandl 2010). The fundamental difference of sex and apomixis in flowering plants mostly lies in the meiotic versus apomeiotic female development. Vegetative propagation does not involve a development from a single-cell stage and is not regarded as a mode of apomictic reproduction but rather as a mode of clonal growth (Mogie 1992).

Apomixis in angiosperms is heritable (Nogler 1984), but the genetic regulatory mechanisms are unexpectedly complex. For gametophytic apomixis, the two components, apomeiosis and parthenogenesis, are under different genetic control and can be uncoupled (Ozias-Akins and van Dijk 2007). In natural systems, apomeiosis is due to temporal or spatial de-regulation of genes controlling the sexual pathway rather than an independent trait (Albertini et al. 2004; Curtis and Grossniklaus 2007; Grimanelli 2012; Grimanelli et al. 2001; Koltunow and Grossniklaus 2003). The differentiation of pre-meiotic cells into megaspore mother cells is controlled by ARGONAUTE proteins via small RNA silencing pathways. AGO9 suppresses gametic cell fate in somatic cells, as Ago9 defect mutants in *Arabidopsis* produce multiple initial cells that are able to undergo gametogenesis (Olmedo-Monfil et al. 2010). AGO104 represses somatic cell fate in the archespore. Thus, AGO proteins apparently specify cell fate for gametophyte development for one up to a few cells in the ovule, but they do not influence meiosis itself. Meiotic and apomeiotic initials can start embryo sac development even in parallel in the same ovule (Hojsgaard et al. 2013). Apomeiotic initials just act as surrogate cells for the meiotic products, or the spores, and ectopic, aposporous cell formation is even dependent on the production of a meiotic tetrad (Koltunow et al. 2011). Apomeiosis and parthenogenesis could result, rather, from epigenetic, possibly silencing actions that are exerted on the normal sexual reproduction pathway by a set of genes that are inherited as a unit. Genetic mapping studies in numerous model taxa have revealed that the loci controlling apomeiosis are located in large non-recombinant regions of the genome (Ozias-Akins and van Dijk 2007). The apospory- or diplospory-specific regions appear as dominant factors in a heterozygous state. So far, attempts to pinpoint “candidate genes” across different apomictic model systems have failed (Ozias-Akins and van Dijk 2007). Apospory-linked loci have apparently evolved convergently in repeat-rich chromosomal regions in both monocot and dicot families (Okada et al. 2011).

Natural apomicts, however, do not show alterations of core meiosis genes, and AGO proteins probably just specify the cells that undergo meiosis but do not influence the cytological processes of meiosis itself that is under different genetic control. In *Arabidopsis*, a combination of three mutants of meiosis-specific genes turns meiosis into a mitosis-like cell division (MiMe system) resulting in unreduced embryo sacs (d’Erfurth et al. 2009). But only the simultaneous suppression of the genes OSD1/TAM, AtSpo11-1, and Atrec8 leads to the production of unreduced male and female gametes. The synthetic MiMe mutants just express apomeiosis and do not exhibit parthenogenesis. Thus, they produce offspring with a doubled chromosome number. The artificial MiME system has not yet been observed in natural apomicts, and no alternations of meiosis genes are yet known. The likelihood that apomixis can be acquired by combinations of mutations in natural systems is extremely low because single mutations alone result in sterility and would be selected against (Van Dijk and Vijverberg 2005).

The second step of apomixis, parthenogenesis, is under an independent genetic control. The transcription factor SOMATIC EMBRYOGENESIS RECEPTOR KINASE1 (SERK1) in *Arabidopsis* probably increases the embryonic potential in ovules and egg cells (Curtis and Grossniklaus 2007). However, the genetic basis of adventitious embryony is much less studied than in gametophytic apomixis.

Land plants never have completely abandoned meiosis but maintained it on the male side without fundamental alterations. On the female side, they have either bypassed or modified it. Facultative sexuality is frequent because, occasionally, sexual development may remain dominant and meiotically reduced egg cells may be produced in parallel to apomictic egg cells (Nogler 1984). Sexual and apomictic pathways can even run in the same ovule and may even be competitive, thus reducing the frequencies of sexual offspring during development (Hojsgaard et al. 2013). In diplosporous plants, facultative sexuality is less frequent because there is no alternative initial cell for embryonic sac development left other than the unreduced megaspore but, still, sexually formed seeds can be observed in large-scale screenings (Aliyu et al. 2010). In general, these occasional sexual events produce sufficient genetic variation in populations to respond to environmental variability (Cosendai et al. 2013; Hörandl and Paun 2007; Lushai et al. 2003; Paule et al. 2011; Paun et al. 2006).

The ubiquity and maintenance of meiosis, even in asexual plants, is striking and a challenge for evolutionary theory. So what is the driving selective force that maintains meiosis in plants?

The main aim of this perspective paper is to refine a theory that DNA restoration is the main function of sex (Hörandl 2009). We will not provide a comprehensive review of development and meiosis but focus on the essential processes during prophase of meiosis I, which we interpret to have the function of repair of oxidative DNA damage. We will first reassess the original hypothesis by the Bernstein group that meiosis originated as a repair mechanism of DNA breaks (Bernstein 1998; Bernstein and Bernstein 1991) and review findings in meiosis research supporting their hypothesis. As a secondary aim, we will review current findings on oxidative stress as the major putative trigger of such DNA lesions; third, we will hypothesize that oxidative stress is the trigger for the onset of meiosis and DSB formation by exploring potential chemical reactions during this process (the oxidative stress initiation hypothesis); fourth, we will discuss a hypothesis as to how the expression of facultative apomixis could be influenced by oxidative stress in extant plants. Finally, we will discuss perspectives and challenges for further research in the field.

## The DNA repair hypothesis

Carol and Harris Bernstein and coworkers were the first to propose a consistent hypothesis that chromosomal crossing over at meiosis might have evolved as a repair mechanism of oxidative double strand DNA damage for which a second homologous, undamaged chromosome is needed as a template (Bernstein 1998; Bernstein and Bernstein 1991; Bernstein et al. 2012; Bernstein et al. 1988). Their ideas stemmed from observations that, in fact, meiosis is not at all optimized to create new gene combinations because Holliday junctions can be resolved with and without crossover. Crossovers result in reciprocal exchanges accompanied by gene conversion, while non-crossovers result in gene conversion of tracts but without reciprocal exchange. Therefore, non-crossovers are quite inefficient in the sense of creating new gene combinations. In fact, the DNA repair hypothesis has gained support from evolutionary studies showing that meiosis-specific proteins are derived from bacterial proteins involved in repair mechanisms of oxidative damage and that mitotic repair proteins are involved in meiosis (Malik et al. 2008; Ramesh et al. 2005). These results suggest that meiosis could have originated as a DNA repair mechanism in early eukaryotes.

The hypothesis of DNA repair as a main force for maintenance of meiosis, however, has not been broadly accepted. The existence of various DNA repair mechanisms besides meiosis (Table 1) has been voiced as a major point of criticism (Birdsell and Wills 2003). Most of these mechanisms do not require a second, homologous chromosome and are mutagenic to a higher extent (see Bleuyard et al. 2006; Friedberg et al. 2006). Homologous recombinational (HR) repair involves a second chromosome and is, thus, regarded as the most accurate and least mutagenic repair mechanism. But HR repair is costly because a second chromosome is essentially required as template. The second homolog can be provided in two ways: (1) via mitotic cell division; and (2) via mixis and the fusion of gametes of two parental individuals. HR repair during mitosis can remove most of the oxidative DNA damage as long as cell divisions are going on in the respective tissues. Mitotic HR repair requires a second chromosome, which is only available with sister chromatids. Thus, mitotic HR repair is *not* available in postmitotic somatic tissues, and a more accurate repair mechanism is needed to avoid mutations. In addition, in the case of mitosis, the sister chromatid can be similarly damaged because it has shared the same level of stress exposure leading to the development of tissue-wide disease phenomena that again may cause DNA damage. This can be avoided if mixis occurred before HR repair. Consequently, the costly meiotic HR repair, which requires mixis at some stage of the life cycle before, is reserved for the immortal germline cells as they represent the initials of the next generation, while for the mortal somatic cells, non-homologous repair mechanisms with higher mutation risks suffice. Somatic mutation accumulation after reproduction is of minor relevance for fitness and further evolution of a lineage (Hörandl 2009, 2013).

**Table 1:** Arguments for and against the ODI hypothesis

Pros	Cons
Oxidative stress is inherent in eukaryotic life because of aerobic respiration and photosynthesis (plants)	Eukaryotes can keep a redox homeostasis with antioxidants
Increased oxidative stress initiates sexual reproduction	Experimental evidence so far available only for fission yeast and algae (*Volvox*); fixed developmental programs in animals
Hydroxyl radical and other ROS can easily arise within the nucleus from hydrogen peroxide because the nucleus contains iron to catalyze the Fenton reaction	ROS chemistry inside the nucleus is quite unknown
Homologous recombinational DNA repair is the most efficient and least mutagenic mechanism	HR repair is also available during mitosis but not in postmitotic tissues
HR repair requires a second chromosome with a different stress history; the likelihood that the same gene is damaged is lower than with sister chromatids; meiotic HR repair uses the second homolog	Permanent diploidy would suffice for HR repair
The meiosis-specific Spo11 protein initiates meiosis; tyrosine has strong antioxidant properties and causes DSBs close to damaged DNA sites	Empirical study needed to confirm that Spo11 binds not randomly, but to previously damaged sites
The repair of DNA lesions via Spo11 results in a double strand break	Chemical reactions during Spo11 activity need to be studied
The DSB is repaired as described, but more frequently resulting in non-crossovers than in crossovers	So far no alternative explanation for the excess of NCOs versus COs
Meiosis is not at all optimized to produce crossovers with efficient recombination	This argument speaks against the hypothesis of recombination as a main function, but does not provide direct evidence that meiosis is optimized for DNA repair
Almost all asexual organisms maintain meiosis in a modified way; ancient asexuals have special DNA repair mechanisms	A minimum of genetic variation is required
Condition-dependent sex is a consequence of stress of an organism that is maladapted	Condition-dependent sex is due to fitness-associated selection

Some authors have argued that permanent diploidy would suffice to provide a homolog for HR repair, but would not require meiosis (Kondrashov 1993). Early eukaryotes were probably haplontic, and they needed mixis to get a second chromosome set. Meiosis was initially perhaps a tool to return to the default haploid stage. Most higher, multicellular eukaryotes have diplontic or diplohaplontic life cycles. In the long run, recessive deleterious mutations accumulate in diploid genomes, and selection will promote meiotic segregation (Otto 2003). Segregation at meiosis allows for a regular return to the haploid stage and thus a more efficient elimination of defect mutants among gametes or gametophytes (Hörandl 2009; Hörandl 2013). These aspects of diploid–haploid cycles, however, do not directly relate to the putative repair functions at the prophase of meiosis I, but rather support a concept that meiosis has various different DNA restoration functions (Hörandl 2009). Some ancient asexual animals (bdelloid rotifers) seem to have colinear chromosomes with special DNA repair mechanisms, which may also reflect a special adaptation to regular desiccation (Fischer et al. 2013; Schon and Martens 2003).

Flowering plants differentiate their germline precursors, the archespore, in the adult, diploid or polyploid sporophyte. Oxidative DNA damage in sporophytes before meiosis can be kept under control by three mechanisms. In the first, plants produce a broad array of secondary metabolites which, in concert with specific enzymes, generate a highly efficient antioxidant system that maintains a homeostasis of ROS (reactive oxygen species) elimination and overproduction (Hadacek et al. 2011; see below). In the second, various mitotic and non-recombinational DNA repair mechanisms are known for plants (Bleuyard et al. 2006). In the third, most natural oxidative stress originates in the photosynthetic organs (usually the leaves), which can be renewed regularly (Foyer and Noctor 2009; Halliwell 2006; Pfannschmidt and Yang 2012).

Under assumptions that oxidative stress is the trigger for the onset of meiosis, a natural increase of oxidative stress is required to initiate flower induction and differentiation of the archespore. In *Arabidopsis*, increased temperatures induce earlier flowering, which is coupled to an increase of the antioxidant enzyme ascorbate peroxidase due to increased levels of hydrogen peroxide, which may contribute to flower induction (Lokhande et al. 2003). For flower development, glutaredoxins using glutathione as cofactor probably play a role for signaling and activation of transcription of genes related to flower development (Li and Zachgo 2009). Prolonged photoperiods have long been known to induce flowering (Amasino 2010; Dennis and Peacock 2007). In temperate to northern regions, both temperature and day length increase in spring. Notably, this is the major flowering period for the great majority of plant species in these regions. In the tropics, seasonal variation in day length and temperature is low, so, in these areas, drought stress induces mass flowering (Sakai et al. 2006). Drought stress, however, can disturb redox homeostasis in a similar fashion (Miller et al. 2010). These types of abiotic stress occur regularly and repeatedly, and the resulting oxidative stress would lead to the accumulation of mutations if only non-homologous DNA repair were to be employed. Meiosis is the more efficient DNA repair mechanism because it relies on a second chromosome that (1) has been confronted with a different stress history and (2) whose DNA damage regions have been repaired repeatedly. Mitosis cannot compete in terms of efficiency.

Many relevant differences between animals and plants play a role here. Oxidative stress derives mostly from respiration, and extensive oxidative damage in muscle cells resulting from motility (Bernstein and Bernstein 1991) cannot be removed by aborting these organs. Protection from high oxidative damage in somatic cells might be one reason why animals differentiate germline cells during very early development from the somatic cell lines. Germ cells are being produced after a division-of-labor principle of female and male gametes as described by Allen (1996), while eggs are being produced early and remain in a resting stage with an inactive pro-mitochondrion that avoids exposure to oxidative stress and allows the inheritance of undamaged female mitochondria. Male gametes need active mitochondria for motility during the fertilization process, and thus, they cannot be kept apart from oxidative stress. Continued production of sperm is perhaps needed to avoid long-term oxidative damage on the nuclei of male gametes. Somatic cells, however, are destined to age and die, so accumulating oxidative damage is tolerable (Allen 1996).

So far there exists no empirical evidence that meiosis relates to frequencies of DNA damage. But it has become evident in the last decades that DSB frequencies during meiosis do not correlate with those of crossovers (Bernstein et al. 1988). More recent research on meiosis supports earlier findings that non-crossovers are, by far, the most frequent outcome of meiosis in fungi and animals (Bernstein et al. 2012). In plants, the crossover frequency is only about 10 per 230 DSB in *Arabidopsis*. Cytological markers suggest a 10- to 40-fold excess of NCO over CO markers; crossover rates are roughly inversely correlated to genome size, which speaks for a control ensuring a minimum number of crossovers rather than an increasing total number of crossovers (De Muyt et al. 2009). The number of crossovers is obviously limited to a narrow range whereby a FANCM helicase may be involved (Crismani et al. 2012). FANCM orthologs are also involved in the regulation of non-crossovers in fission yeast, which points to an evolutionary highly conserved mechanism (Lorenz et al. 2012). In yeast, an average of 90.5 crossovers to 66.2 non-crossovers was estimated, which fits well to estimates of 140–170 DSBs at meiosis. In non-crossovers, gene conversion alone even might have a homogenizing effect on allele diversity. That is, c. 40 % of DSBs do *not* increase, but rather reduce genetic diversity. Crossovers and non-crossovers are not evenly distributed over the genome, and DSB frequencies are not strictly congruent with recombination hotspots along chromosomes. No significant association of crossovers and non-crossover regions to certain sequence motifs or gene ontology terms could be detected (Keeney 2008).

Bernstein et al. (2012) suggest that repair mechanisms can be subdivided into two major pathways: a few double-Holliday junction events, resulting in crossovers used for proper chromosome segregation, and many synthesis-dependent strand annealing events, resulting in non-crossovers used for unprogrammed double strand damages. That is, DNA damages of various types could be converted into DSBs as a “common currency” for various repair mechanisms and purposes. This model, however, does not provide the basis for an explanatory model for the initiation of double strand breaks that we propose here (see below). We do not assume DSBs as the primary cause but as a consequence of oxidative damage repair.

Frequent NCOs do not support the classical hypotheses that selective forces for recombination in offspring could maintain DSB formation and crossovers during meiosis I in order to increase genetic variation in offspring. Chiasmata are required for correct segregation because they provide physical connections between homologous chromosomes during the first meiotic division. Strand invasion is required for chromosome pairing and synapsis at meiosis (Cifuentes et al. 2010; De Muyt et al. 2012; Lorenz et al. 2012; Page and Hawley 2004; Wilkins and Holliday 2009). However, one crossover per chromosome would suffice to serve this purpose (Cifuentes et al. 2010; Crismani et al. 2012; Lorenz et al. 2012). It appears inappropriate to cut DNA on 140–170 sites only to repair it afterward. Wilkins and Holliday (2009) suggest that crossovers originated initially to limit erroneous recombination events, but this does not explain why so many DSBs occur that do not result in crossovers. Furthermore, recombination is a consequence, not a causal explanation, of meiosis. Crossovers are important for establishing the physical connection of chromosomes (synapses) for correct segregation (De Muyt et al. 2009).

Segregation and production of haploid meiotic products may have a selective advantage only in diplontic or diplohaplontic organisms. After a prolonged diploid phase, recessive mutations accumulate as they are effectively masked by the unmutated site of the homologous chromosome. The return to the haploid phase after meiosis unmasks these mutations and increases the efficacy of purging selection against deleterious mutants in haploid gametes or gametophytes (Hörandl 2009, 2013; Schubert 2011). Since early eukaryotes were most likely haplontic organisms, the mechanisms of DSB formation must have originated before the shift to diplontic or diplohaplontic life cycles. Proteins involved in DSB formation and those in non-crossover regulation are highly conserved among eukaryotes (Lorenz et al. 2012).

The formation of DSBs at the prophase of meiosis I and the subsequent repair via resection, synthesis, and ligation thus appears to be a risky investment for a minimal chance to gain a selective advantage. Therefore, DSB formation and formation of Holliday junctions could have a primary function other than recombination. Recombination is probably just a side effect of this process (Hörandl 2009). Admittedly, rejection of recombination-based hypotheses does not prove that the function of meiosis is repair (Table 1).

## The oxygen paradox: oxidative stress versus environment-relating signaling

**Fig. 1:**
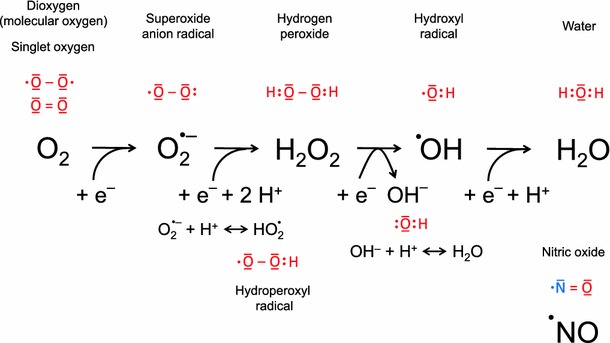
Reactive oxygen and nitrogen species (ROS and RNS). Four electrons are required to reduce oxygen formally to water, an integral reaction of respiratory chemistry in mitochondria. Thereby, reactive and toxic intermediaries, superoxide anion radical, hydrogen peroxide and hydroxyl radical can arise. In the cell, this chemistry is under tight control; superoxide dismutases convert O_2_^·−^ into H_2_O_2_, which catalases and peroxidases reduce into water. Low concentrations of ROS are important for cell signaling; higher ones are toxic because they can damage cell components (oxygen paradox). Conversely, specific enzymes, such as NADPH oxidases, can specifically catalyze ROS formation. Nitrogen can undergo a similar chemistry, and nitric oxide resembles a RNS. In chloroplasts, relaxing chlorophylls activate triplet (molecular) into singlet oxygen, which becomes reduced much more easily because of its activated state

Photosynthetic cyanobacteria can oxidize water to molecular oxygen and caused a significant increase in atmospheric oxygen concentration 750 mya. This change in the atmospheric gas composition led to a large mass extinction that affected all those prokaryotes that took their energy from oxidizing hydrogen sulfur or iron salts (Hartman 1996). Aerobic respiration basically is an oxidative breakdown of organic molecules for gaining energy in the form of ATP equivalents and, for this purpose, is a magnitude more efficient than anaerobic respiration that uses sulfur, methane, or hydrogen as electron acceptors. The reduction of oxygen provides the largest free energy release per electron transfer among all elements of the periodic system (Catling et al. 2005). As a consequence, aerobic pro- and eukaryotes evolved that tolerated substantial concentrations of molecular oxygen in their environment. The formation of molecular oxygen in the oxidation of water (photosynthesis) and water in the reduction of molecular oxygen (respiration) depend on the exact transfer of four electrons. Unscheduled one-electron transfers, however, may lead to the formation of superoxide anion radical (O_2_^·−^), hydrogen peroxide (H_2_O_2_), and hydroxyl radical (^·^OH), all reactive oxygen species (ROS) responsible for oxygen toxicity (Fig. 1). Free radicals are chemical species possessing one or more unpaired electrons and capable of independent persistence (Fridovich 1998; Halliwell 2006; Pierre and Fontecave 1999). The paradox of aerobic life, or the “Oxygen Paradox,” is that eukaryotic aerobic organisms cannot exist without oxygen. Concomitantly, oxygen is inherently toxic to them (Davies 1995; Davies 2000).

Electron transport chains contribute to the correct oxidative cleavage of water to oxygen and hydrogen and the correct reduction of oxygen to water in chloroplasts and mitochondria. In both processes, accidental one-electron reductions of oxygen lead to the formation of O_2_^·−^ radicals (Foyer and Noctor 2009). H_2_O_2_ is formed by dismutation of two O_2_^·−^ molecules and further reduction generates ^·^OH (Fenton reaction), which has a half-life of 1 nsec and is one of the fastest and most universal oxidation reagents in nature (Hadacek et al. 2011; Halliwell 2006; Møller et al. 2007). H_2_O_2_ is a more long-lived ROS (1 μs) and can permeate cell organelles and even whole cells. The kinetics of these one-electron transfers is affected by the presence of transition metal catalysts, especially iron, which is the most abundant, while copper, nickel, cobalt, and manganese are more scarce (Frey and Reed 2012).

To control the damage that might be afflicted by oxidative ROS chemistry, an antioxidative defense system has evolved that is comprised of enzymes such as superoxide dismutase (SOD), catalases (CAT), peroxidases (APX), and low molecular weight metabolites including ascorbic acid and glutathione (Foyer and Noctor 2005). In a concerted way, it aims to maintain redox homoeostasis in cells of unicellular and multicellular organisms. In strong contrast to the toxic effects of higher ROS amounts, low ROS concentrations are essential for signaling environmentally induced stress such as excess light, drought, salinity, low temperatures, ozone, wounding, pathogen, and herbivore attack. Conversely, ROS have been recognized as important upstream signaling components with effects on Ca^2+^ signaling, MAP kinases, hormones, gene expression, protein modification, and downstream effects on stomata closure, gravitropism, programmed cell death, predator and parasite resistance, growth, and morphology (del Rio and Puppo 2009; Mithöfer et al. 2004; Pfannschmidt and Yang 2012; Ślesak et al. 2007). The advantage of ROS involvement in signaling is, thus, accompanied by a fundamental constraint: oxidative damage of vital molecules, such as lipids, proteins, and DNA, that has to be repaired continuously and efficiently. The oxygen chemistry provides aerobic organisms with a superior signaling system that facilitates survival and reproduction in earlier life stages and contributes to death in later stages. The inclusion of prokaryotic endosymbionts, which later became cell organelles, i.e., the mitochondria, and the plastids in plants were key innovations for eukaryotic metabolism (Margulis and Sagan 1991).

## ROS damage and its repair

**Fig. 2:**
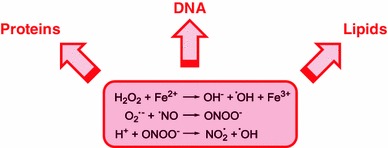
Hydroxyl radical-generating chemical reactions in tissues exposed to oxidative stress that damage DNA, proteins, and lipids

Hydroxyl radical is the most damaging ROS and can arise by various routes. The most prominent is the Fenton reaction, the mostly ferrous iron-catalyzed reduction of H_2_O_2_. The damaging potential in biological systems is reflected by the attention directed to such reactions in the development of many human diseases (Kell 2010; Stohs and Bagchi 1995). But other routes to ^·^OH are also possible. A prominent reactive nitrogen species is nitric oxide (^·^NO), a nitrogen radical species (RNS) that, similar to ROS, is regarded as an important signaling molecule (Bellin et al. 2013; Neill et al. 2003). It can react with superoxide anion radical to peroxynitrite. At a physiological pH, peroxynitrite easily protonates to peroxynitrous acid that can undergo hemolytic fission to form ^·^OH and nitric dioxide radical (NO_2_^·^), a further powerful oxidizing agent, among others (Halliwell 2006). Evidence exists that oxidative damage occurs on all molecules that are present in the cell, lipids, proteins, and DNA (Fig. 2) for which direct and indirect repair mechanisms have evolved (Davies 1995, 2000; Moldovan and Moldovan 2004; Møller et al. 2007).

ROS can oxidize amino acids. Carbonylation, the oxidation into reactive aldehyde or ketone groups, leads to protein inactivation, crosslinking, or breakdown (Rinalducci et al. 2008; Sweetlove and Møller 2009). Peptides formed during breakdown of oxidized proteins, though, have been suggested to constitute important secondary signal components facilitating a specific gene expression response in answer to a specific stress (Møller and Sweetlove 2010). One of the most studied oxidation of biomolecules is that of lipids and lipid peroxidation (Halliwell and Gutteridge 2007).

While DNA is vital to cell division and survival, it also is a victim of various kinds of oxidative damage that can affect the base, the deoxyribose sugar, and the phosphodiester moiety (Friedberg et al. 2006). Estimates exist suggesting that, even under normal physiological conditions, 1 base modification occurs in 130,000 bases in nuclear DNA, in mitochondrial DNA, and even in 8,000 bases. The types of DNA damage include strand breaks (single and double), sister chromatid exchange, DNA–DNA and DNA–protein cross-links, and base modifications. Both purine and pyrimidine bases can be oxidized, though cytosine and especially thymine appear to be most sensitive (Wagner et al. 1994). The loss of aromaticity destroys the required planarity and causes distortions of the DNA double helix. Damage of the DNA backbone, the deoxyribose and the phosphodiesters, may result in strand breaks (Davies 2000; Halliwell and Gutteridge 2007; Pratviel et al. 1995). In congruence with the high probability of DNA damage, several pro- and eukaryotic enzymes repair oxidatively damaged DNA by both direct and excision repair mechanisms (Friedberg et al. 2006). Accordingly, evolution of efficient repair mechanisms of oxidative damage of DNA represents a substantial constraint of aerobic eukaryotic life. Early eukaryotes have taken over the enzyme machinery for DNA repair from bacteria (Ramesh et al. 2005; Malik et al. 2008), but the mechanics of DNA repair had to be improved. In a circular prokaryotic genome, recombination between inverted repeats may cause inversion, while recombination between directed repeats may split the ring-shaped genome into two parts and lead to subsequent loss of one of the products if segregation into daughter cells is not concerted (Schubert 2011). Linear chromosomes in eukaryotes allow for homologous recombinational repair of double strand breaks during mitosis and meiosis.

## The oxidative damage initiation hypothesis for maintenance of meiosis

Assuming ROS-induced DSBs of DNA as a direct cause for meiosis represents a major problem for the original Bernstein hypothesis. Spontaneous DSBs are the least frequent form of DNA lesion in extant organisms, and the assumption of severe and regular DNA breaks to initiate meiosis is unrealistic in light of efficient ROS scavenging systems. The natural situation in extant, multicellular organisms would be rather an endogenous, moderate excess of ROS after increased photosynthetic or respiratory activity (Foyer and Noctor 2009) that will cause many minor DNA lesions. Here we will present a hypothetical redox chemical model as to how the meiosis-specific protein Spo11 may become activated as an efficient scavenger of such minor oxidative damages.

**Fig. 3:**
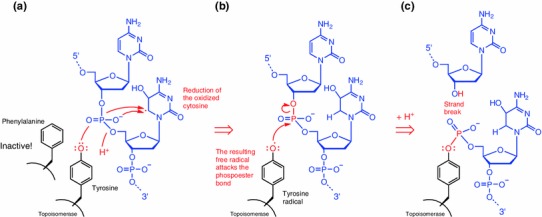
Redox-chemical formation of Spo11–DNA adduct. The catalytic tyrosine residue of Spo11 becomes so oxidized by oxidatively damaged DNA that it reduces in the fashion of an antioxidant. For example, cytosine is shown. Similar oxidative damage is possible on all four DNA bases: the resulting tyrosyl radical than attacking the phosphodiester bond initiating a DSB. The reaction scheme purposely ignores any metal coordination complex formation that most probably occurs in the nucleus. Transition metals, such as iron, copper, or manganese can act as catalysts of this electron transfer. The reaction is shown for one strand only

Recombination of homologous maternal and paternal chromosomes occurs during prophase I stage in most sexually producing organisms and consists of DSB formation and repair (Keeney 2001). The protein Spo11 serves as catalyst and is thought to act via a topoisomerase-like reaction to form a transient, covalent protein–DNA intermediate. After DSB formation, Spo11 becomes removed from the DNA and the 5′ termini are resected to yield variable length, 3′ single-stranded tails. In a series of reactions that depend on homologs of the bacterial RecA protein, intact homologous tails undergo strand invasion that ultimately generate mature recombinant products, as is shown in yeast (Keeney 2008). *Arabidopsis* has two homologs, Spo11-1 and Spo11-2, which both require catalytically active tyrosine residues to cause DSBs: mutants in which the active tyrosine, was replaced by the structurally closely related amino acid phenylalanine, which lacks a phenolic hydroxyl group, failed to initiate DSBs (Hartung et al. 2007). This finding strongly supports our proposed redox chemical model of Spo11 activation as it pinpoints the hydroxyl function as essential component for the protein’s activity. However, for activation of the hydroxyl group, a free radical must be present in the vicinity (Fig. 3).

Tyrosine is a phenolic amino acid. Plant phenols, flavonoids, and phenolic acids have been long renowned for their antioxidant properties (Hadacek et al. 2011; Rice-Evans et al. 1996). They are able to scavenge free radicals such as ^·^OH (1).1


Flavonoids and other plant phenols are known to protect against oxidative DNA damage, but their mode of action not only includes scavenging of free radicals, but also chelation of transition metals that catalyze oxidative chemistry. The formation of phenolic radicals usually results in their polymerization (Hadacek et al. 2011; Surh 1999; Valko et al. 2006).

In resemblance to the oxidation of a phenolic antioxidant in the scavenging process (1), we propose that a similar redox chemistry occurs when the catalytic tyrosine residues come into close vicinity of a oxidatively damaged DNA region (Fig. 3): when free radicals are present in the DNA, the two tyrosine residues become oxidized (Fig. 3a) and, as tyrosyl radicals, attack the phosphoester bridges of the DNA deoxyribose backbone (Fig. 3b). As a consequence, a DSB is initiated at the sugar-phosphate backbone near the DNA region that has become oxidatively damaged, while a protein–DNA complex is formed (Fig. 3c), as proposed by Edlinger and Schlögelhofer (2011). Figure 3 only shows one of many possible damage scenarios, but the chemical principle remains the same. Polymerization of tyrosine is hindered as it is part of a macromolecule that adjusts the tyrosine residues as gripping pliers for the DNA double strand.

The chemistry outlined in Fig. 3 is less complex than probably occurs in the nucleus. Electron transfer rates in redox chemistry are influenced strongly by the presence of transition metal catalysts, such as iron. For example, see the Fenton reaction (Fig. 2). A recent finding certainly supports our proposed mechanism of electron transfers for Spo11 DSB initiation; the plant cell nucleolus was identified as an abundant iron pool in healthy dividing cells (Roschzttardtz et al. 2011).

The hypothetical mechanism of Spo11 DSB initiation is central to the oxidative damage initiation hypothesis (ODI) for meiosis and supports the argument that meiosis serves as a repair mechanism of damaged DNA (Bernstein and Bernstein 1991; Bernstein et al. 2012; Bernstein et al. 1988). The amino acid tyrosine is known to contribute to substrate specificity of other enzymes as well, e.g., cyclooxygenase (Schneider et al. 2007). In contrast to the original hypothesis, which assumed the pre-existence of breaks, we assume that minor, ROS-induced DNA lesions can induce Spo11 activity; the DSB formation is just a consequence of the repair function of Spo11. In addition, the ODI hypothesis offers a possible explanation of how the repair mechanism may become targeted specifically to oxidatively damaged DNA regions. So far, no general mechanism has been suggested that determines the site of DSB initiation by Spo11 on the chromosome apart from chromatin opening by histone modification (Edlinger and Schlögelhofer 2011). However, this also could be caused by protein oxidation.

Our model is at the present stage of research quite hypothetical and might be much more complex than shown here. A couple of other proteins are essential for DSB formation, and they interact with Spo11 during DSB formation. However, many of these proteins do not share sequence homology across different groups of eukaryotes, and therefore, it is difficult to develop a general model for the protein interaction during DSB formation. In plants, also AtPRD1-3 homologs are essential for DSBs, but interaction of these proteins with Spo11 and the chemical reactions during DSB formation are quite unknown (Edlinger and Schlögelhofer 2011).

Repair of oxidative lesions by Spo11 appears to be a risky mechanism because it causes DSBs. Therefore, it is reasonable that Spo11 activity is restricted to meiosis where a second, homologous chromosome is always available. Homologous recombinational repair is, by far, the most accurate mechanism for repair of DSBs (Bleuyard et al. 2006). The ODI hypothesis is in accordance with the restoration theory that a costly repair mechanism in multicellular organisms is only useful for germline cells or spores but not for mortal somatic cells (Hörandl 2009). In contrast, the generally maintained view is that sex increases genetic variation and, thereby, promotes evolutionary adaptation, an idea that traces back to August Weismann (Weismann 1904). Goddard and coworkers (Goddard et al. 2005) claimed to provide experimental support for Weismann’s hypothesis by showing that a sexual yeast strain population that differed from the asexual just by the ability to undergo meiosis, propagated more efficiently than the asexual under harsh environments. Likewise, this effect could also be caused by meiosis because it is the more efficient repair mechanism of oxidative DNA damage that was caused by these harsh environmental conditions. If an organism is maladapted to an environment, as postulated by the FAS hypothesis, the observed investment into sexual reproduction can be interpreted either as a fitness-associated response to selection or as a direct effect of stress on that organism.

The ODI hypothesis purports filtering out drastic mutations and contributing such to species’ robustness and identity (Heng 2007) to be the evolutionary benefit of meiosis and sex. It does not contradict the results of the Goddard group’s experiments, but it does provide an alternative explanation by suggesting that the benefit of sex is not primarily increasing genetic variance in the offspring but contributing to DNA repair in attempts to prevent impairment of the offspring.

Under natural conditions, environmental stress with a moderate increase of photosynthesis and respiratory activity is likely to cause shifts in the redox homeostasis as a trigger for meiosis (Foyer and Noctor 2009). For plants, increased photoperiods regularly induce flowering as they occur naturally in spring in temperate regions and drought periods or cyclones in tropical regions. Shifts in oxidative stress levels may be responsible in all scenarios. It remains questionable whether the artificial treatments of plants with H_2_O_2_, nitrogen or compressed air, as performed by Kelliher and Walbot (2012), can actually simulate oxidative stress development as caused by natural stress factors. The authors report inhibition of germ cell specification in maize anthers after artificial oxidizing treatments and suggest that hypoxia triggers meiotic cell fate. Moreover, a biochemical assessment of actual redox status in cells, and of meiosis protein activity, is still lacking for this system. Support for the direct influence of a more natural environmental stress on the mode of reproduction is available from a couple of studies on facultative apomictic plants. This will be outlined in the next section.

## Oxidative stress and the expression of apomixis

Several experimental approaches directly or indirectly suggest a positive correlation of oxidative stress to sex in facultative apomictic plants. In the facultative sexual/asexual green alga *Volvox carteri*, sex is a response to increased levels of heat stress (Nedelcu et al. 2004; Nedelcu and Michod 2003). In this species, heat stress causes the production of a 30 kDa glycoproteic inducer protein (SI). This protein stimulates the gonidia to produce egg- or sperm-bearing sexual spheroids. The fusion of gametes results in the formation of a desiccation-resistant, hibernating zygospore, which germinates and undergoes meiosis when favorable conditions return in the next spring. In this organism, sexual development in the gonidia is triggered by an approximately twofold increase of reactive oxygen species after heat stress, and it could be demonstrated that actually ROS activate two sex genes, the *SI* gene and the *clone B* gene. ROS could have been activated by these genes because antioxidant catalases decreased their transcript level. The formation of the zygospore most likely represents a response to increased oxidative stress, and meiosis is the mechanism of recombinational DNA repair before the next haploid generation is formed. Nedelcu and Michod (2003), Nedelcu et al. (2004) suggested that sex might be one alternative stress response in addition to cell-cycle arrest and apoptosis.

**Fig. 4:**
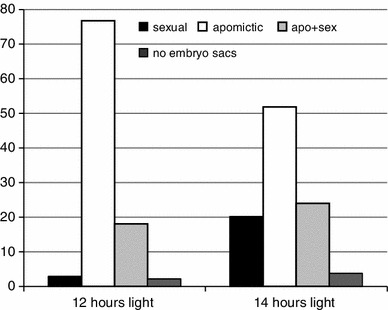
Frequencies of modes of embryo sac formation in *Paspalum* (Poaceae: Panicoideae) under normal light conditions (12 h) and under light stress (14 h); data from Quarin (1986)

Several experimental studies (see Fig. 4) show that prolonged photoperiods (light stress) significantly increase meiotic frequencies compared to aposporous embryo sac in facultatively apomictic/sexual flowering plants (Evans and Knox 1969; Gupta et al. 1969; Knox 1967; Quarin 1986; Saran and Dewet 1976). Currently, there is no biochemical study available to explain these phenomena. However, light stress and increased photosynthetic activity are major sources for ROS overproduction with effects on coordinated gene expression (Pfannschmidt 2003; Pfannschmidt and Yang 2012). Accordingly, shifts in redox homeostasis may act as functional triggers for sexual development.

Some indirect hints for sexual reproduction correlating with redox homeostasis have been suggested by comparative gene expression studies. In sexual/asexual *Boechera* taxa, a relative of *Arabidopsis*, the sex-specific SuperSAGE tags from microdissected ovules show a significant increase of oxidoreductase gene activity during the premeiotic to the meiotic stage. Genes that were overall significantly over-represented in meiotic stages in sexual plants compared to apomictic plants include those of glutathione metabolism, flavone and flavonol biosynthesis, terpenoid biosynthesis, and phenylpropanoid biosynthesis (Sharbel et al. 2010). The latter secondary metabolite classes are usually associated with biotic stress, but their accumulation also might prove as beneficial to survive abiotic stress (Hadacek et al. 2011). These findings support a hypothesis that expression of meiosis versus apomeiosis may be related to regulation of oxidative stress and actual ROS levels within ovules. To develop an integrative hypothesis for the observed phenomena, three points have to be considered: (1) the male meiosis; (2) the genetic control of apomixis; and (3) indirect effects of polyploidy.

1. In almost all apomictic plants, microsporogenesis is still present and runs without major alterations (Asker and Jerling 1992). Many authors report disturbances of chromosome pairing and segregation and formation of unbalanced gamete formation that often results in partly aborted pollen (Hörandl et al. 1997; Izmailow 1996; Podio et al. 2012). But some reduced, functional pollen is usually being produced, and there is no fundamental change in the functionality of meiosis or in male development.
2. Apomeiosis in the female development is probably under an independent genetic control from meiosis genes themselves. Regulatory pathways inactivation leads to an ectopic or heterochronous expression of developmental genes, affecting somatic traits in the gametic cell lineage (Grimanelli 2012). Proteins of the ARGONAUTE family play a key role in cell fate determination: AGO9 triggers gametogenesis (embryo sac development) and, in *AGO9* mutants, produces multiple embryo sacs out of somatic cells similarly as in apospory (Olmedo-Monfil et al. 2010). AGO104 proteins trigger somatic development and, in *AGO104* mutants, turn meiosis into a mitosis-like division, similar to that in diplospory (Singh et al. 2011). The deregulation of these genes is probably an effect of hybridization and heterochronicity (Grimanelli et al. 2005; Sharbel et al. 2010). Determination of gametophytic cell fate in aposporous or diplosporous initials does not necessarily directly influence the genetic control of meiosis proteins. In natural apomicts, initiations of meiosis and sporogenesis follow in principle the same genetic control as in obligate sexual plants. The sexual pathway is just being suppressed by the parallel apomictic pathway, which is initiated synchronously (apospory) or subsequently (diplospory). Grimanelli (2012) postulates that a cell-to-cell signaling mechanism must be activated or repressed in sporogenic tissues. Both small RNA pathways (Boyko and Kovalchuk 2011) or retrotransposons (Grimanelli 2012) have been pointed out as candidates for this function.
3. Most apomictic plants are polyploids, which alter physiology, increase amounts and composition of secondary metabolites, photosynthesis rates, stress tolerance, and may buffer effects of oxidative stress and deleterious mutations by having more sets of chromosomes in the nucleus (te Beest et al. 2012). However, they also increase photosynthetic electron carrier capacity (Coate et al. 2012). Both hybridization and polyploidy alter quantitative and qualitative composition of secondary metabolites (Orians 2000), which also may contribute to maintaining of the redox homeostasis in plant tissues (Hadacek et al. 2011). We build our model on the assumption that polyploids maintain cell ROS homeostasis more efficiently than related diploids.

Polyploidy further affects the cytological mechanisms of meiosis because correct pairing and segregation of chromosomes is demanding in a doubled chromosome set. Allopolyploids usually exhibit a more regular meiosis than autopolyploids. Meiotic aberrations are commonly observed in newly formed polyploids, but selection against infertile plants likely acts for re-diploidization (Cifuentes et al. 2010). For this selection process, male meiosis results in four meiotic products while female meiosis usually results only in one functional megaspore, so it is much more likely that an alternative asexual pathway succeeds on the female side than on the male side.

**Fig. 5:**
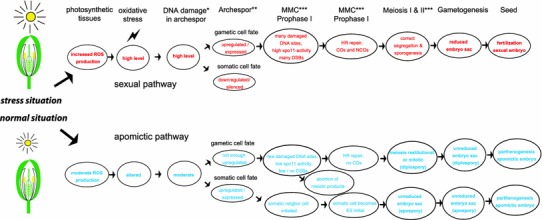
Hypotheses of the effects of light stress on photosynthesis, oxidative stress, functionality of meiosis, and increased frequencies of sexual embryo sac formation in apomictic flowering plants.* Single asterisk* Assuming a ROS-mediated signal transfer between tissues;* double asterisk* after (Grimanelli 2012); *trible asterisk* see Fig. 3 for detailed model of Spo11 activation as oxidative damage scavenger; further processing of meiosis as described (Bernstein et al. 2012; De Muyt et al. 2012). *MMC* megaspore mother cell

We propose the following model for the stress-sensitivity of sexual reproduction (Fig. 5): In diploid, obligate sexual plants, prolonged photoperiods cause mild oxidative stress, which is signaled from green parts of the plants to reproductive tissues via ROS. This trigger initiates flower induction and tissue differentiation in floral organs and in the archespore where ARGONAUTE proteins act to differentiate megaspore and microspore mother cells from the surrounding somatic tissues by suppressing the somatic cell fate (i.e., mitotic divisions). Mild oxidative stress in the reproductive tissues increases the levels of DNA damage in the megaspore mother cells that oxidize Spo11 tyrosyl residues (Fig. 3). Such DSB formation is triggered as a direct and unavoidable consequence of oxidative damage. The meiosis machinery repairs the DSBs via homologous recombinational repair, as described (e.g., Bleuyard et al. 2006), with a few crossovers required for correct chromosome pairing and correct segregation at anaphase I.

**Fig. 6:**
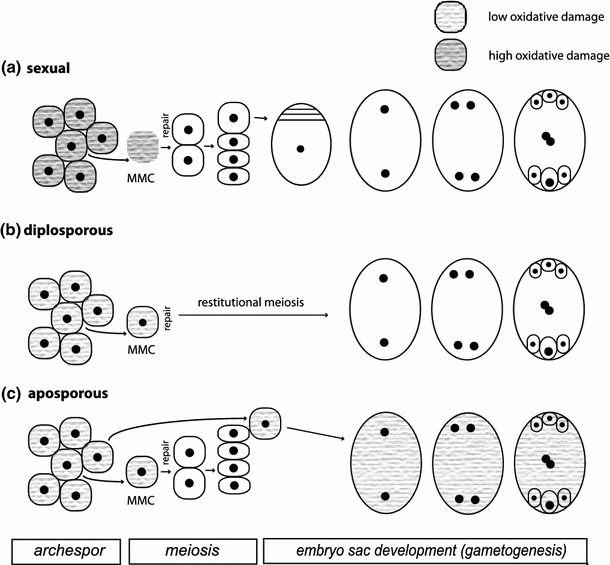
Hypothesis on the effect of oxidative stress on the formation of megaspores from megaspore mother cells (MMCs) versus apomictic initials of embryo sac formation

Apomictic polyploids with an improved oxidative stress regulation in vegetative tissues might not suffer sufficient oxidative damage to trigger Spo11 activity and DSB formation, resulting in partly abortive meiotic phenotypes. If, at the same time, apospory and diplospory arise in some, but not all, polyploids the alternative apomictic pathway succeeds with seed formation as a surrogate for the sexual pathway (Fig. 6). This model is consistent with observations of facultative sexuality in almost all apomictic plants. If oxidative stress is artificially increased, as in Quarin’s experiments (Fig. 4), then proportions of regular meiotic products and sexually formed embryo sacs increase at the expense of the apomictic ones.

## Directions for future research

Some evidence supports our view that meiosis originated not only for DNA restoration, but also for oxidative damaged DNA repair. Assuming this, we have to regard allelic recombination as only a by-product of this process that does have other important evolutionary consequences yet is not the reason why sex is maintained. The restoration hypothesis, in contrast, provides an explanatory model for maintenance of obligate sex because of this fundamental function (Hörandl 2009).

The reasoning of ODI hypotheses is derived from observations of quite different research fields: redox chemistry, physiology, DNA function and repair, meiosis and chromosome research, developmental pathways, and evolutionary history. To gain more robust support and to grow into a challenging null hypothesis for the generally accepted ones, further relevant information is needed from all these fields. The main aim of this perspective paper is to stimulate broader interdisciplinary thinking and develop integrative research projects to test the different aspects redox chemistry, meiosis, and apomixis specifically.

Redox chemistry is a relatively young field, and it is still a challenge to reliably trace and measure reactions and results of oxidative damage on organic molecules. Most ROSs are extremely unstable with msec half-lives and, thus, are difficult to measure in living tissues (Bonini et al. 2006). ROS may undergo various different reactions, not only with DNA and RNA molecules, but also with other cellular molecules; ROS activity does not necessarily cause linear effects. Therefore, careful experimental work is required in attempts to fine-tune administering oxidative stress levels in tissues in simulation of natural environmental conditions. For plants, quantifying oxidative stress and DNA damage in the archespore under different environmental conditions (e.g., different photoperiods) will be fundamental to understand the postulated correlations.

Most importantly, the role of oxidative stress and ROS on the onset and signaling pathways during the prophase of meiosis I need further investigations. DNA repair of oxidative damage has become a major field of research and has facilitated the recognition of the functional and evolutionary origin of meiosis out of DNA repair mechanisms. Nevertheless, the role of the meiosis-specific enzymes during meiotic recombination in extant, modern eukaryotes is still not well understood. Most authors take it for granted that DSBs at meiosis are done “on purpose” by Spo11. We propose a chemical model of how Spo 11 might recognize oxidatively damaged DNA sites. The big challenge is to study the chemistry of one functional group on a macromolecule that contains a plethora of them. Recombination hotspots need to be investigated as to whether they represent damaged sites (not necessarily breaks, but also lesions on the bases), as hypothesized here. Further studies on meiosis proteins and chromosome behavior under different levels of oxidative stress are needed to understand the postulated repair functions of meiosis. Female versus male meiosis and developmental pathways need to be studied, and meiotic and apomeiotic development needs to be compared.

The classical model plants like *Arabidopsis* and maize do not exhibit natural apomixis, and thus, no alternative to sex can be explored with experimental treatments. While low oxidative stress even might inhibit flowering (Lokhande et al. 2003), high oxidative stress might have severe damaging effects on tissues. Facultative apomictic plants could serve as model systems for investigating effects of environmentally induced oxidative stress on expression of modes of meiosis. However, the issue of whether oxidative stress has direct influence on shifts from obligate sex to apomixis needs further investigation. For applications of apomixis in agriculture, it needs to be considered whether an incomplete suppression of meiosis might be unstable under different environmental conditions, which makes the trait less useful for commercial applications. A complete, genetically engineered mutant-based approach like the MiMe system may provide environmentally insensitive, obligate apomixis which would reliably fix certain genotypes. But the lack of meiotic DNA repair may have detrimental effects on genomes of the offspring after a few generations and thus exert negative effects on vigor and fertility of plants. It would be useful, therefore, to integrate meiosis and apomixis research for a comprehensive understanding of processes acting during development.
